# Optimization of the Blocking and Signal Preservation Protocol in High‐Parameter Flow Cytometry

**DOI:** 10.1002/cpz1.70214

**Published:** 2025-09-25

**Authors:** Oliver T. Burton, James Dooley, Adrian Liston

**Affiliations:** ^1^ Department of Pathology University of Cambridge Cambridge United Kingdom; ^2^ These authors contributed equally to this work

**Keywords:** Fc receptor, flow cytometry, immune phenotyping, intracellular staining, spectral flow cytometry

## Abstract

High quality input data is the key to successful interpretation of any scientific assay. In flow cytometry, fluorescently‐conjugated antibodies allow us to simultaneously measure an incredible range of protein‐based targets on single cells with a high degree of specificity. Limiting the quality of data generated, however, is the non‐specific interaction that can occur between antibodies and off‐target binders. Judicious use of blocking reagents can improve the specificity of the staining by reducing this non‐specific binding to cells, improving the sensitivity of the assay to detect the authentic signal above assay noise. Additional beneficial effects include preventing interactions between dyes and even limiting the degradation of dyes, improving data quality. In this article, we provide a workflow for minimizing these unwanted effects, increasing specificity and sensitivity in highly multiplex flow cytometry. © 2025 The Author(s). Current Protocols published by Wiley Periodicals LLC.

**Basic Protocol 1**: Surface staining

**Basic Protocol 2**: Intracellular staining

**Basic Protocol 3**: Intracellular cytokine staining

## INTRODUCTION

In this article, we provide an optimized, general‐use approach to blocking non‐specific interactions and signals. We aim to provide a generalizable protocol that is suitable for use in most high‐parameter flow cytometry assays involving human or murine cells. Additionally, we provide examples of the beneficial or detrimental effects observed when using common blocking reagents.

The incredible specificity of antibody binding via variable domains permits precise, sensitive measurement of proteins and other molecules in flow cytometry. Many other interactions are possible, however, particularly once the antibodies are conjugated to fluorophores (Burn et al., 2024; Francis et al., 2017; Konecny et al., 2024; Kristensen et al., 2021; Smith et al., 2025), and while these events may occur with much lower affinity, in aggregate their contributions can greatly reduce or otherwise compromise the sensitivity of staining in cytometry assays. Blocking these non‐specific interactions can enhance the signal to noise ratio, improving sensitivity, provided that the blocking reagents are used appropriately and with attention to the potential for introducing new undesirable effects.

A particular problematic interaction is with the Fc receptors. Fc receptors provide a natural binding partner for immunoglobulins, independent of the variable domain specificity, and are of particular concern for immunologists due to the prevalence of receptor expression in the hematopoietic system. The low‐affinity Fc receptors CD16 and CD32 have dissociation coefficients around 10^−6^ molar, and typically require aggregation of multiple IgG molecules around the antigen in order to increase the avidity of the interaction between the Fc and Fc receptors to the point that biologically relevant binding occurs (Wang et al., 2022). Among the high‐affinity Fc receptors, only CD64 (FcγRI) is likely to meaningfully impact high‐parameter flow cytometry assays, which rely on monoclonal IgG antibodies for staining, rather than IgE or IgA. The amount of Fc‐mediated binding can be expected to depend on a complex interplay of Fc receptor expression by cell type and activation status, as well as the specific isotypes and host species of the antibodies used for staining. For example, most anti‐mouse monoclonal antibodies for flow cytometry derive from rat, with rat IgGs generally exhibiting reduced interactions with mouse Fc receptors (compared with mouse IgG to mouse FcγR). By contrast for human targets, mouse antibodies are most frequently used, which bind well to human FcγR (Wang et al., 2022), increasing non‐specific binding potential.

Whereas Fc‐mediated binding increases background binding on certain cell types, other non‐specific binding events such as dye interactions can occur in a cell‐independent manner. For example, Brilliant dyes, NovaFluors, and Qdots are all prone to dye–dye interactions (Konecny et al., 2024; Team, 2017, 2021; Thermo Fisher, 2023), potentially leading to increases in signal when multiple reagents in a family are used simultaneously. While less common than Fc‐binding, these dye–dye errors can be more insidious, as they create skews in the representation of signals not just up and down within a marker, but can manifest as signals in different markers, due to the interaction producing a correlated emission pattern. Understanding how to mitigate these effects is critical to reproducibility in high‐parameter flow cytometry where many reagents must be used together.

Finally, certain classes of dyes, particularly those comprised of multiple fluorophore molecules, a.k.a. tandems, are susceptible to conversion into their constituent parts. This breakdown can result in erroneous signals for the constituent fluorophore(s) rather than the original tandem molecule. In high‐parameter flow, the base fluorophore—or one spectrally similar to it—will almost certainly be present in the assay as well, so these breakdown signals will be misassigned to an alternative marker, resulting in biological misinterpretation. In many cases, appropriate handling of the fluorophore‐conjugated reagents and samples stained with tandems can mitigate these effects. In this article, we try to provide a generalizable protocol that in many cases will reduce these effects through reagents selection, sample preparation, and panel design. The blocking steps in this protocol have been tested on both standard short (20 to 60 min) staining incubations and in overnight staining approaches (Whyte et al., 2022).

## STRATEGIC PLANNING

Determine the host species of the directly conjugated antibodies in your panel. For best results in blocking non‐specific binding, obtain normal sera from the same species as the antibodies being used (e.g., rat normal sera, if staining mouse samples with largely rat antibodies). Avoid using serum from the same species as the cells being stained if you are staining for immunoglobulins (e.g., IgG, IgM) as this will either limit staining or cause erroneous staining.

For smaller cell numbers, halving the volumes of blocking and staining solutions may be appropriate.

For panels that do not contain SIRIGEN “Brilliant” or “Super Bright” polymer dyes, Brilliant Stain buffer may be omitted, however the polyethylene glycol (PEG) in the buffer also reduces non‐specific binding of many non‐Brilliant fluorophores in human samples where the donor has been immunized with PEG‐containing vaccines, e.g., SARS‐CoV‐2 vaccination (Team, 2021).

For panels that contain NovaFluors, CellBlox (Thermo Fisher) must be used. We do not cover that use case in this article as we have been unable to optimize the usage of this reagent adequately (see Commentary section).

*CAUTION*: Fixatives mentioned in this article contain paraformaldehyde and should be used with appropriate safety considerations in a fume hood.

*CAUTION*: Sodium azide is highly toxic. Follow appropriate guidelines for handling according to the safety data sheet.

*NOTE*: All protocols involving animals must be reviewed and approved by the appropriate Animal Care and Use Committee and must follow regulations for the care and use of laboratory animals. Appropriate informed consent is necessary for obtaining and use of human study material.

## Basic Protocol 1 SURFACE STAINING

This protocol provides an optimized, general use approach for reducing non‐specific interactions in high‐parameter flow cytometry when only surface staining is being performed.

### Materials

Mouse serum (Thermo Fisher, cat. no. 10410; various sources are acceptable)
Rat serum (Thermo Fisher, cat. no. 10710C; various sources are acceptable)
Tandem stabilizer (BioLegend, cat. no. 421802)
Cells (details provided here will be most relevant for mammalian immune cells)
Brilliant Stain Buffer (Thermo Fisher, cat. no. 00‐4409‐75) or BD Horizon Brilliant Stain Buffer Plus (BD Biosciences, cat. no. 566385)
FACS buffer (see recipe)

Sterilin clear microtiter plates, 96‐well V‐bottom (Fisher Scientific, cat. no. 1189740)
Centrifuge
20‐ and 200‐µl multichannel pipettes and tips
Flow cytometer

1 Prepare a blocking solution comprised of rat serum, mouse serum, tandem stabilizer, and serum from any other host species (Table 1).

**Table 1:** Blocking Mix Preparation

Reagent	Dilution factor	Volume (µl) for 1‐ml mix
Mouse serum	3.3	300
Rat serum	3.3	300
Tandem stabilizer	1000	1
Sodium azide (10%)*^a^*	100	10
FACS buffer	Remaining volume	389

^*a*^ Sodium azide may be omitted for short‐term use.

2 Dispense cells into V‐bottom, 96‐well plates for staining.
  Cell numbers should be standardized to reduce batch effects.
3 Centrifuge 5 min at 300 × *g*, 4°C or room temperature, and remove supernatant.
4 Resuspend cells in 20 µl blocking solution.
5 Incubate 15 min at room temperature in the dark.
6 Prepare surface staining master mix (Table 2), if not already prepared.
  We suggest using up to 30% (v/v) Brilliant Stain Buffer.

**Table 2:** Surface Staining Master Mix Preparation

Reagent*^a^*	Dilution factor	Volume (µl) for 1‐ml mix
Tandem stabilizer	1000	1
Brilliant Stain Buffer	3.3	300
Antibody 1	As appropriate	
Antibody 2	As appropriate	
FACS buffer	Remaining volume	

^*a*^ Sodium azide may be added for long‐term storage. Brilliant Stain Buffer Plus may be used in place of Brilliant Stain Buffer, reducing the volume by 4×.

7 Add 100 µl surface staining mix to each sample and mix by pipetting.
  Details of the antibodies used in the mix are up to the user.
8 Incubate 1 hr at room temperature in the dark.
9 Wash with 120 µl FACS buffer.
10 Centrifuge 5 min at 300 × *g*, 4°C or room temperature. Discard supernatant.
  If your panel requires multiple surface staining steps, include those here.
11 Repeat the wash and centrifugation, steps 9 to 10, with 200 µl FACS buffer.
12 Resuspend samples in FACS buffer containing tandem stabilizer at 1:1000 dilution.
13 Acquire the samples on your cytometer.

## Basic Protocol 2 INTRACELLULAR STAINING

If you intend to stain for markers inside the cell using antibody‐based reagents, you will likely benefit from an additional blocking step prior to intracellular staining. Permeabilization of the cell after fixation exposes a much larger range of epitopes for antibodies to interact with, so in many cases a blocking step after permeabilization and before intracellular staining can improve specificity, and thus, the signal‐to‐noise ratio.

### Additional Materials (also see Basic Protocol 1)

Perm/Wash buffer (BD, cat. no. 554723)
ddH_2_O
Cytofix/Cytoperm fixation and permeabilization solution (BD, cat. no. 554722) or Foxp3/Transcription Factor staining buffer set (eBioscience, cat. no. 00‐5523‐00)
Fume hood

1 Prepare double the amount of blocking mix (Table 1).
2 Prepare Perm/Wash buffer by diluting according to the manufacturer's instructions (usually 10× with ddH_2_O).
3 Perform the surface staining protocol (Basic Protocol 1) until step 12, but instead of resuspending in FACS buffer containing tandem stabilizer, resuspend in the fixative of your choice. Use 100 to 200 µl fixative. Perform this step in the fume hood.
  For general purpose immune phenotyping, we recommend either the BD Cytofix/Cytoperm kit or the Thermo Fisher Foxp3/Transcription Factor kit.
4 Incubate with fixative for 20 to 30 min in the dark, usually at 4°C.
5 Centrifuge 5 min at 600 × *g*, 4°C. Dispose of supernatant according to the safety data sheet for the fixative.
6 Wash twice with Perm/Wash buffer. For plates, add 200 µl Perm/Wash buffer, spin 5 min at 600 × *g*, 4°C, discard supernatant, and repeat.
7 Add 20 µl blocking mix to the cells and resuspend gently.
8 Incubate 15 min at 4°C in the dark.
9 Prepare intracellular staining master mix.
10 Add 100 µl intracellular staining master mix (user determined). Pipette up and down gently to mix.
11 Stain 1 hr at room temperature in the dark or overnight at 4°C in the dark.
  Overnight staining will usually improve detection of intracellular targets.
12 Wash twice with Perm/Wash buffer.
13 Resuspend in FACS buffer containing tandem stabilizer (1 in 1000).
14 Acquire on cytometer.

## Basic Protocol 3 INTRACELLULAR CYTOKINE STAINING

While largely similar to the Intracellular Staining protocol (Basic Protocol 2), optimal detection of intracellular cytokines requires additional considerations. To start, you should optimize your stimulation to evoke cytokine production and accumulation in the cell. As this has been extensively covered elsewhere (Lamoreaux et al., 2006; Lovelace & Maecker, 2018; Mair & Tosevski, 2014), we do not address this in the current protocol. In the context of this protocol, we suggest that the choice of fixative is critical to prevent leakage of cytokines during the fixation/permeabilization step and recommend using one of the following: (1) BD Cytofix/Cytoperm, (2) BioLegend Fixation Buffer, or (3) Thermo IC Fixation Buffer. Finally, to prevent cytokine leakage during surface staining steps, we suggest the inclusion of Golgi network inhibitors during the blocking and staining steps.

### Additional Materials (also see Basic Protocols 1 and 2)

1000× monensin solution (BioLegend, cat. no. 420701; various sources are acceptable)
1000× brefeldin A solution (BioLegend, cat No. 420601; various sources are acceptable)
Fixation Buffer (BioLegend, cat. no. 420801), Cyto‐Fast Fix/Perm Buffer Set (BioLegend, cat. no. 426803) or IC Fixation Buffer (Thermo Fisher, cat. no. FB001)

1 For intracellular cytokine staining, add brefeldin A and/or monensin to the surface blocking mix (Table 4).
2 After stimulation, spin down cells for 5 min at 300 × *g*, 4°C or room temperature, in a V‐bottom 96‐well plate and discard the supernatant.
3 Resuspend cells in 20 µl blocking solution.
4 Incubate 15 min at room temperature (or 4°C if preferred) in the dark.
5 Prepare surface staining master mix (Table 5), if not already prepared.
  We suggest using up to 30% (v/v) Brilliant Stain Buffer.
6 Add 100 µl staining master mix (user determined, Table 5). Pipette up and down gently to mix.
7 Incubate 1 hr at room temperature in the dark.
  This length of incubation works well for activation markers. If you are only staining for type‐defining and/or viability markers, we suggest reducing the staining time to 20 to 30 min.
8 Wash twice.
9 Incubate with fixative for 30 min in the dark, usually at 4°C. Perform this step in the fume hood.
  We recommend using BD Cytofix/Cytoperm or BioLegend's Fixation Buffer for most purposes.
10 Centrifuge 5 min at 600 × *g*, 4°C. Dispose of supernatant according to the safety data sheet for the fixative.
11 Wash twice with Perm/Wash buffer. For plates, add 200 µl Perm/Wash buffer, spin 5 min at 600 × *g*, 4°C, discard supernatant, and repeat.
12 Add 20 µl blocking mix to the cells and resuspend gently.
13 Incubate 15 min at 4°C in the dark.
14 Prepare intracellular staining master mix as per Table 3.

**Table 3:** Intracellular Staining Master Mix Preparation

Reagent*^a^*	Dilution factor	Volume (µl) for 1‐ml mix
Tandem stabilizer	1000	1
Brilliant Stain Buffer	3.3	300
Antibody 1	As appropriate	
Antibody 2	As appropriate	
Perm/Wash buffer	Remaining volume	As required to reach 1 ml

^*a*^ Sodium azide may be added for long‐term storage. Brilliant Stain Buffer Plus may be used in place of Brilliant Stain Buffer, reducing the volume by 4×.

**Table 4:** Blocking Mix for Cytokine Staining Preparation

Reagent	Dilution factor	Volume (µl) for 1‐ml mix
Mouse serum	3.3	300
Rat serum	3.3	300
Tandem stabilizer	1000	1
Sodium azide (10%)	100	10
Monensin (2 mM) or brefeldin (2 mg/ml)	1000	1
FACS buffer	Remaining volume	388

**Table 5:** Cytokine Master Mix Preparation

Reagent*^a^*	Dilution factor	Volume (µl) for 1‐ml mix
Tandem stabilizer	1000	1
Monensin (2 mM) or brefeldin (2 mg/ml)	1000	1
Brilliant Stain Buffer	3.3	300
Antibody 1	As appropriate	
Antibody 2	As appropriate	
FACS buffer	Remaining volume	As required to reach 1 ml

^*a*^ Sodium azide may be added for long‐term storage.

15 Add 100 µl intracellular staining master mix (user determined). Pipette up and down gently to mix.
16 Stain 1 hr at room temperature in the dark or overnight at 4°C in the dark (recommended).
17 Wash twice with Perm/Wash buffer.
18 Resuspend in FACS buffer containing tandem stabilizer (1 in 1000).
19 Acquire on cytometer.

## REAGENTS AND SOLUTIONS

### FACS buffer

1 L of 1× PBS (Current Protocols, 2006)
25 ml fetal bovine serum (FBS) (TICO Europe, cat. no. FBS‐EU500; various sources are acceptable)
4 ml of 0.5 M EDTA, UltraPure, pH 8.0 (Thermo Fisher, cat. no. 15575020)
Mix and store up to 2 weeks at 4°C
For longer term storage, add 10 ml of 10% (v/v) sodium azide (0.1% v/v final; see recipe)
  Bovine serum albumin (BSA) may be substituted for FBS. In this case, add 5 g BSA to reach 0.5% (w/v) final.

### Sodium azide, 10%

10 g sodium azide (Sigma Aldrich, cat. no. S2002‐5G)
100 ml ddH_2_O
Store up to 10 years at room temperature

## COMMENTARY

### Critical Parameters

**Figure 1:**
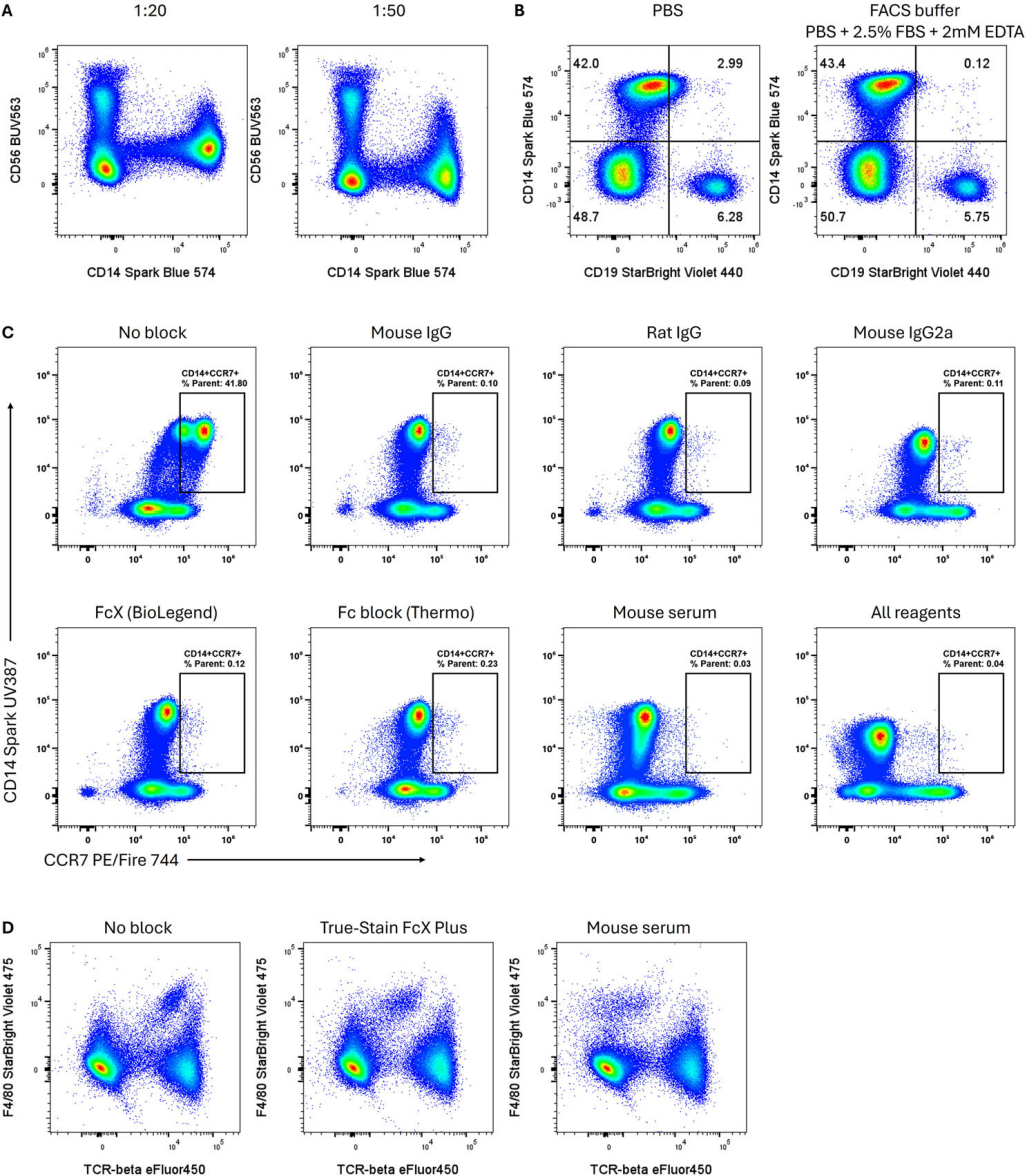
Titration, protein, and immunoglobulins form the base for effective signal presentation. (**A**) Optimizing antibody concentration reduces non‐specific binding. Human PBMCs were stained for 1 hr in the absence of Fc blocking reagents with anti‐CD56‐BUV563 at the indicated concentrations. (**B**) Protein and EDTA reduce non‐specific binding of anti‐CD19 to human PBMC CD14+ monocytes. (**C**) Impact of various FcγR‐blocking reagents on non‐specific binding of anti‐CCR7‐PE/Fire 744. Human PBMCs were fixed, permeabilized, and stained overnight with a highly supra‐optimal concentration of anti‐CCR7. (**D**) Comparison of anti‐CD16/32 Fc block and mouse serum for reduction of anti‐TCRβ binding to mouse splenic macrophages. Gating in all cases is on viable single leukocytes.

Titration should be the first step in reducing non‐specific binding (Bonilla et al., 2024; Burn et al., 2024). In Figure 1A, simply reducing the concentration of anti‐CD56 antibody decreases off‐target binding to CD14^+^ monocytes, improving the separation between positive and negative. Titration should be performed with appropriate blocking. If the blocking conditions need to be modified, the titrations should be redone under the new staining conditions for best results.

In most cases, “fluorescence minus one” (FMO) controls provide good guidance as to where the background should be in the absence of non‐specific binding. Thus, when investigating the specificity of a given antibody, we recommend testing in the context of the panel staining structure that will be used. For example, in a panel on human peripheral blood mononuclear cells (PBMCs), including basic gating markers, such as CD3, CD4, and CD14, will allow the researcher to distinguish between overall non‐specific binding (occurring on both T cells and monocytes) and more Fc gamma receptor‐mediated binding (happening mainly on monocytes and other non‐T cell populations).

**Figure 2:**
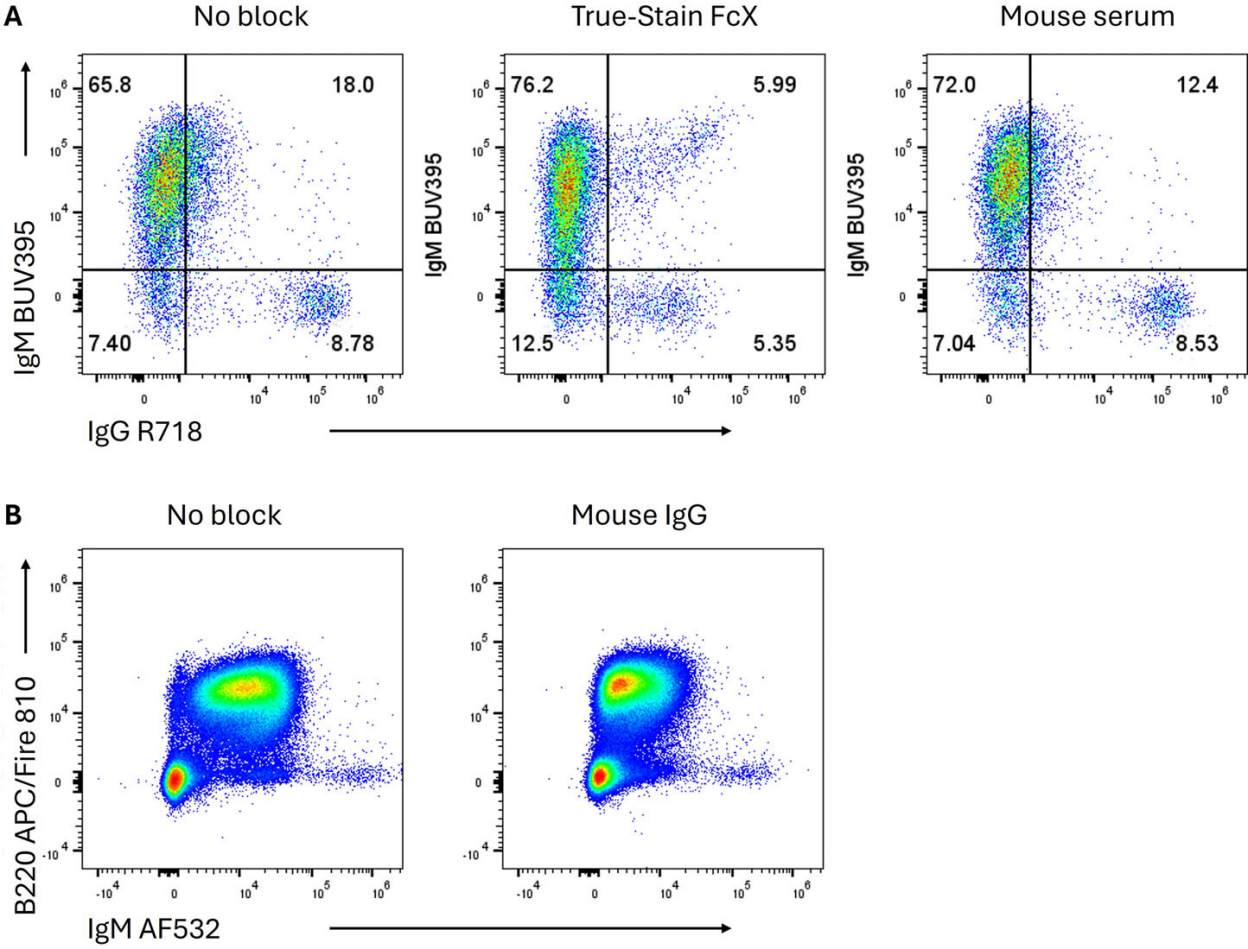
Fc‐blocking reagents can have undesirable effects. (**A**) Staining of human CD19^+^ B cells for human IgG and IgM. When TruStain FcX is added, an artefactual IgG^+^IgM^+^ population appears. (**B**) Staining of murine splenocytes shows a reduction in murine IgM staining on B220^+^ B cells when a preparation of mouse “IgG” is added to the staining mix.

Non‐specific binding of conjugated antibodies can be blocked effectively by using unlabeled non‐specific antibodies from the same host species as the staining reagents (e.g., using mouse serum to block human samples, when most antibodies being used to stain are murine in origin). Serum contains high concentrations of immunoglobulins and is generally a cost‐effective option. In Figure 1C, we provide comparisons of the efficacy of serum versus commercial Fc blocking reagents for mouse and human immune cell staining. We caution the reader to consider carefully whether any of the reagents will neutralize or otherwise distort the specific binding of the conjugated antibody in their assay. For example, commercial human Fc blocking reagents may contain human IgG, and in our experience can reduce on‐target staining for IgG while creating abnormal positivity in Fc receptor‐bearing cells (Fig. 2A). Similarly, mouse serum and certain preparations of mouse immunoglobulins may interfere with staining for IgM and IgG on murine B cells (Fig. 2B).

**Figure 3:**
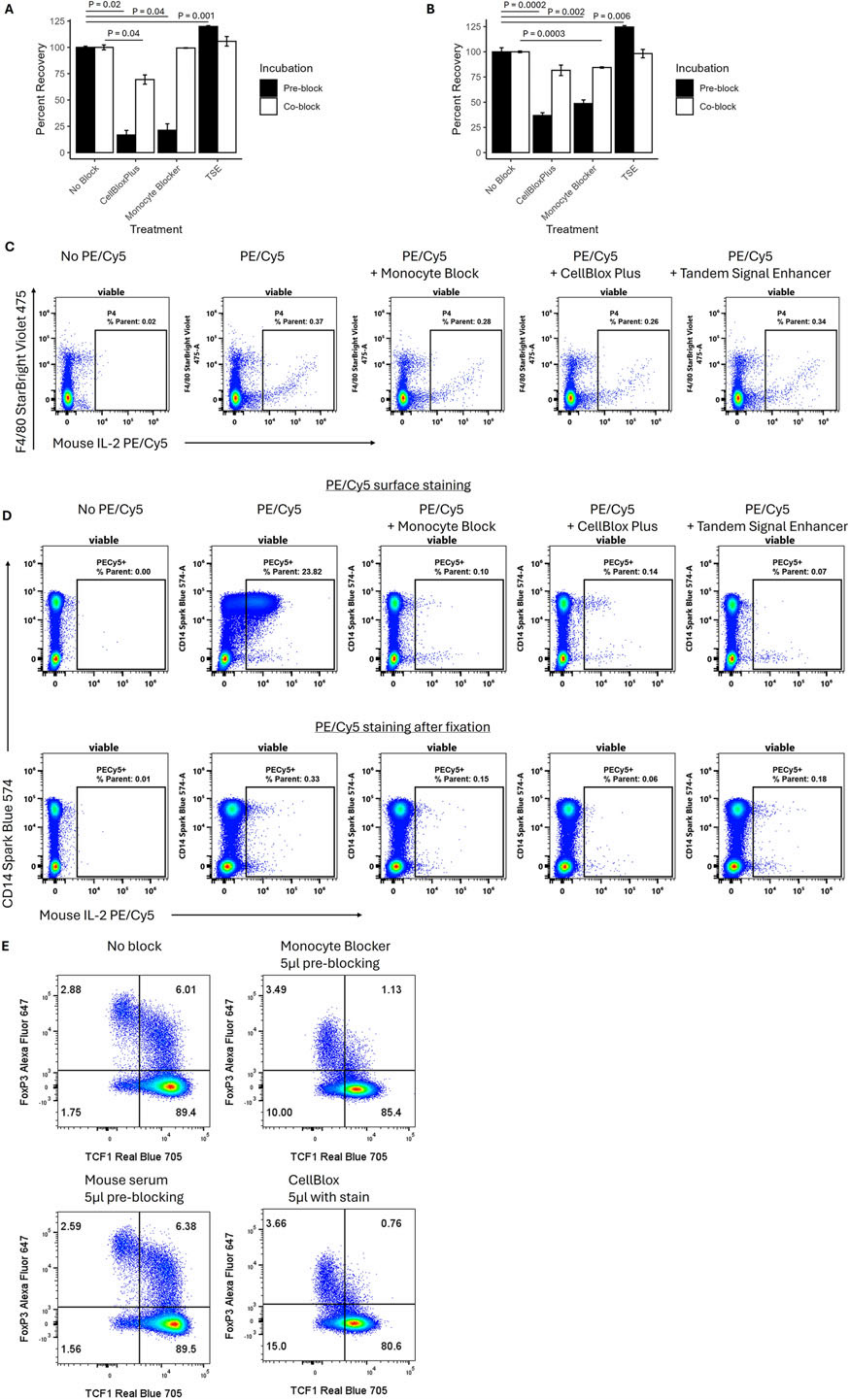
An investigation of beneficial and detrimental effects of monocyte blocking reagents. (**A**) Cell recovery of murine splenocytes after treatment with monocyte blocking reagents either prior to or coincident with antibody staining. (**B**) Cell recovery of human PBMCs. Statistical analysis in (**A**) and (**B**) by *t*‐test with BH correction for multiple hypothesis testing. (**C**) Negligible impact of monocyte blocking reagents on non‐specific binding of anti‐mouse IL‐2‐PE/Cy5 to the surface of unstimulated murine splenocytes. Co‐staining with F4/80 to identify macrophages is shown. (**D**) Non‐specific binding of anti‐mouse IL‐2‐PE/Cy5 to human CD14^+^ monocytes can be inhibited using monocyte blockers or simply by fixing the cells prior to staining. (**E**) The inclusion of monocyte blocking reagents in the intracellular staining incubation reduces transcription factor detection. Human PBMCs are shown with an overnight incubation, gated on CD3^+^CD4^+^ viable T cells.

A second commonly used class of blocking reagents attempts to reduce non‐specific binding of cyanine tandem dyes to monocytes and other myeloid cell types (Jahrsdorfer et al., 2005; Kristensen et al., 2021). Many of these reagents markedly reduce cell recovery, especially if used in a traditional pre‐incubation blocking step prior to staining (Fig. 3A‐B). While we have not found any evidence that certain cell types are lost more than others, which would lead to biased results, an exhaustive analysis is not feasible. If these reagents are used, they should be included in the antibody staining cocktail, not in a separate pre‐blocking step. We note that Milteyni's Tandem Signal Enhancer does not appear to reduce cell recovery, unlike the others we have tested. For murine cells, we find no evidence of any benefit to using these reagents (Fig. 3C) and do not recommend doing so. For human cells, all monocyte blocking reagents were similarly effective in our tests. We find, however, that fixation also largely eliminates non‐specific binding of cyanine tandem dyes to human monocytes (Fig. 3D). As such, incorporating cyanine tandems into a post‐fix staining step may provide an effective and cheaper strategy (Whyte et al., 2022). Exposure to fixatives contributes to cyanine tandem breakdown, so staining with these dyes after fixation can have additional benefits.

In the process of testing these monocyte blocking reagents, we discovered what we suspect is a previously unknown phenomenon. When monocyte blocking reagents are included in intracellular staining protocols, detection of transcription factors is markedly reduced (Fig. 3E). We are uncertain as to the cause but given that the non‐specific binding is already abolished by the fixation step, we recommend avoiding the use of monocyte blocking reagents after fixation and permeabilization.

**Figure 4:**
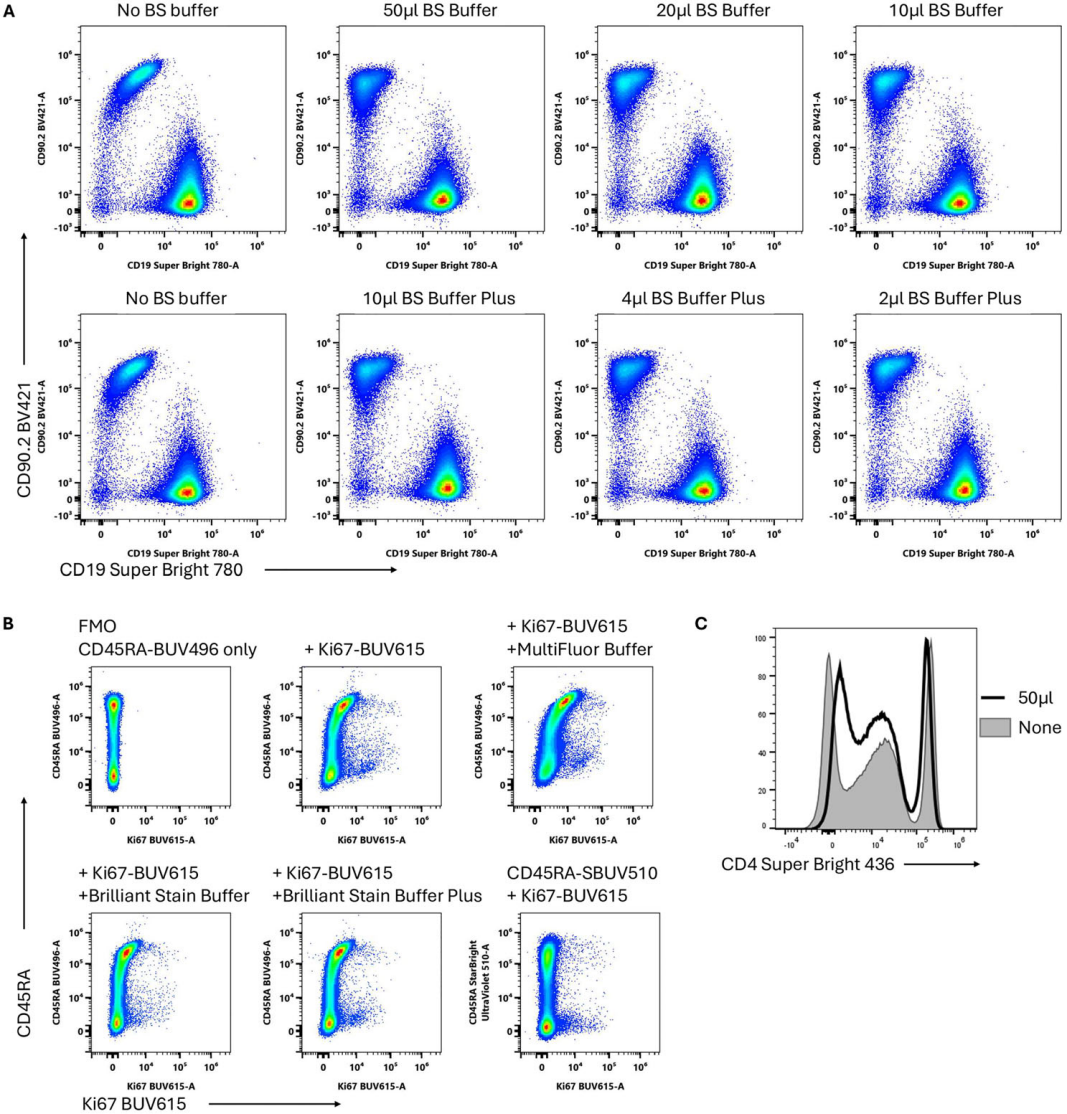
Effective use of Brilliant Stain Buffer. (**A**) Titration of Brilliant Stain Buffer and Brilliant Stain Buffer Plus on murine splenocytes stained with anti‐CD90.2‐BV421 and anti‐CD19‐Super Bright 780. Buffers were added to the antibody cocktails at the point of preparation. Samples were stained for 1 hr at 4°C. (**B**) Solutions for reducing Brilliant UV dye interactions with highly expressed markers. Human PBMCs were stained overnight for CD45RA and Ki67 in the presence of buffers intended to block Brilliant dye interactions. Examples are shown gated on CD3^+^CD4^+^ viable T cells. (**C**) Increased background fluorescence in the Super Bright 436 channel on human PBMCs stained with anti‐CD4 Super Bright 436 in the presence of Brilliant Stain Buffer.

The SIRIGEN polymer family of Brilliant and Super Bright dyes are widely used in high‐parameter flow cytometry due to their relatively narrow and bright emission characteristics. It is well known that these dyes, particularly the ultraviolet dyes, can interact with each other non‐specifically, creating characteristic inward‐curving staining patterns (Fig. 4A). These interactions require two or more such dyes to be present, and do not occur with all conjugates to the same extent in our experience. In most cases, the addition of Brilliant Stain Buffer or Brilliant Stain Buffer Plus can limit these effects to an acceptable level (Fig. 4A). The effects are likely to be most prominent when using UV dyes and when using high concentrations of conjugated antibodies and/or staining molecules that are highly expressed. In Figure 4B, we provide an example of staining human PBMCs with anti‐CD45RA‐BUV496 overnight. While the Brilliant Stain and Brilliant Stain Plus buffers provided modest improvements, in this case, the interaction could not be blocked completely. Swapping the CD45RA from a Brilliant conjugate to an alternative dye provided a solution in this instance, as one of the interaction partners was removed. We note, as others have pointed out previously (Ferrer‐Font et al., 2020), that Brilliant Stain Buffer is fluorescent and can increase the background in portions of the spectrum where BV421 and Super Bright 436 emit (Fig. 4C). We therefore recommend titrating these buffers to determine the optimal amount for the panel in question. We generally use these buffers at half to a quarter the recommended amount and find that if the interactions are not adequately reduced, further optimization of antibody concentrations or panel design is more fruitful than increasing the amount of buffer.

**Figure 5:**
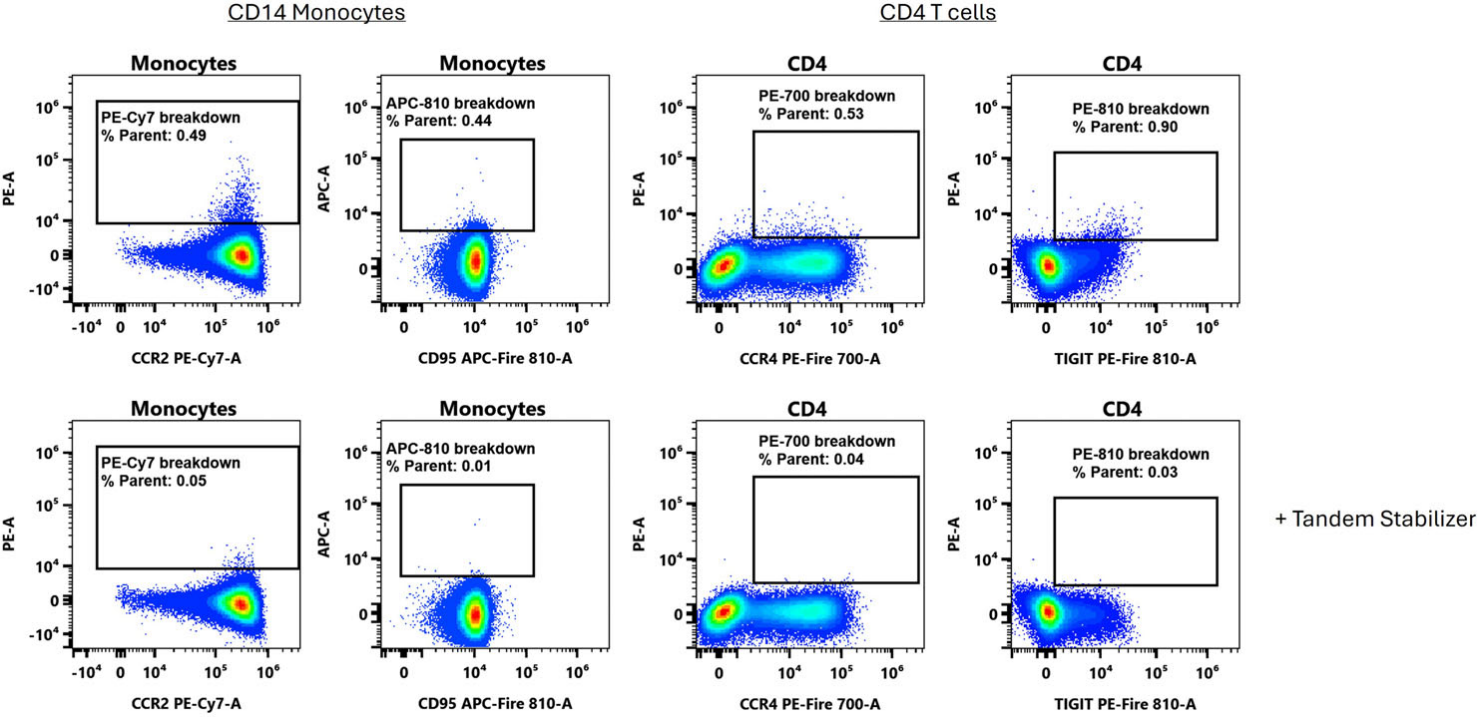
Reduced tandem breakdown with the Tandem Stabilizer. Human PBMCs were stained for 1 hr with cyanine tandem dyes. The PE and APC channels were unmixed, but no PE^–^ or APC^–^ conjugated antibodies were used. The tandem stabilizer was included in all steps of the protocol.

As mentioned previously, cyanine tandem dyes are prone to breakdown. While many of the dyes used in flow cytometry are FRET pairs between multiple fluorophores and can be considered tandems, colloquially in the field “tandems” are considered to be cyanine‐family dyes conjugated to protein‐based fluorophores (PerCP, PE and APC). These tandems can degrade, breaking the linkage between the FRET partners and creating an unwanted increase in signal from the protein‐based fluorophore (for example, PE‐Cy7 degrades to produce more PE signal). This can happen in response to light, heat, fixatives and cellular metabolic activity (Hulspas et al., 2009; Jensen et al., 2020; Le Roy et al., 2009; Morawski et al., 2019). The tandem stabilizer reagent from BioLegend reduces tandem breakdown from PerCP, PE and APC‐based tandems (Fig. 5 and data not shown). In the example in Figure 5, the cells have not been stained with either PE or APC, so all signal in those channels derives from tandem breakdown and is noticeably reduced when the tandem stabilizer is included. We have not observed any unwanted effects of using this reagent.

**Figure 6:**
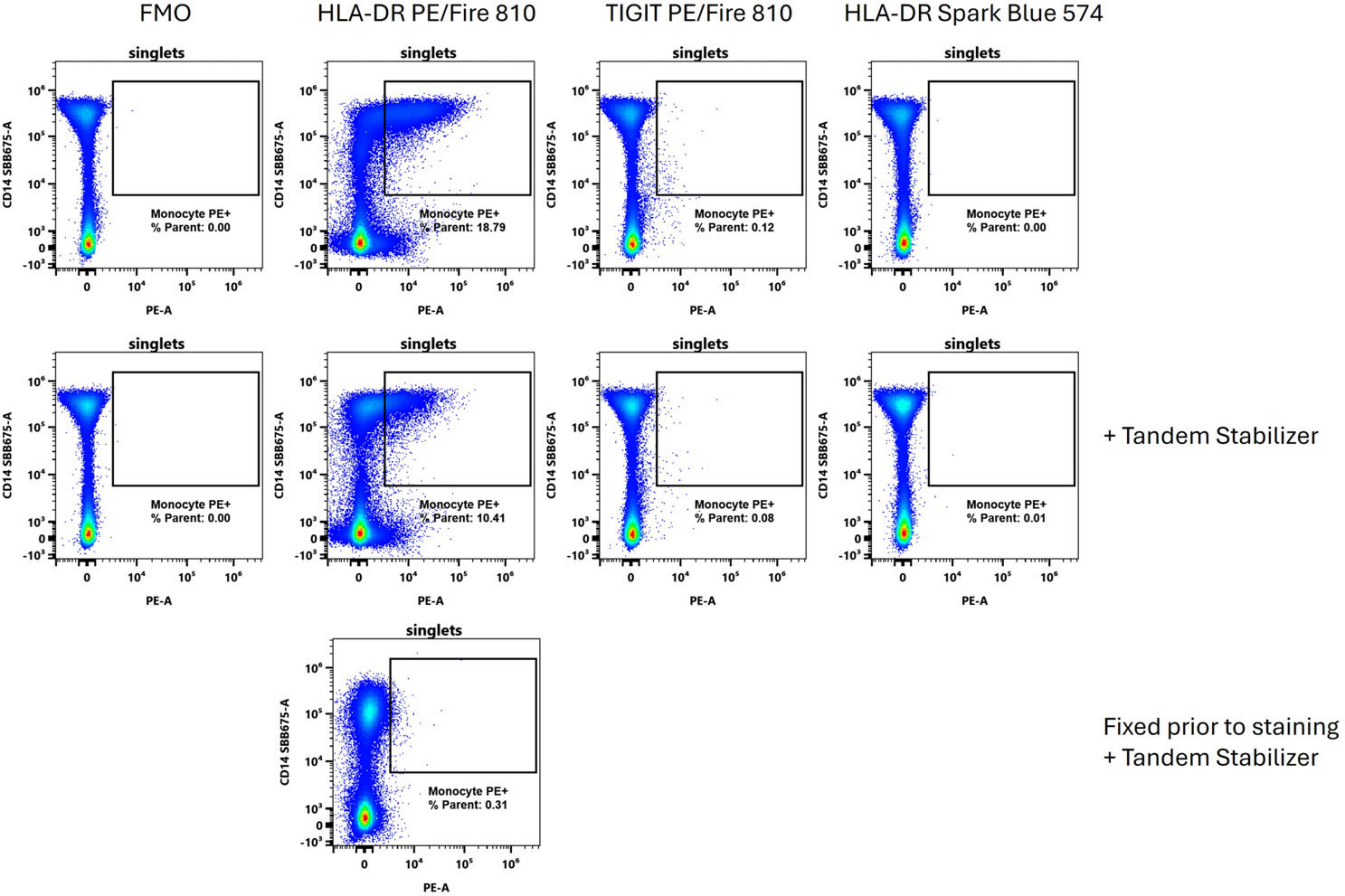
Tandem breakdown can be ameliorated with tandem stabilization, panel design, or fixation. Human PBMCs were stained for CD14 to identify monocytes, and the PE channel was unmixed in the absence of any PE staining, so signal in the PE channel represents tandem breakdown or spillover spread. Samples were stained variable with either anti‐HLA‐DR PE/Fire 810, anti‐TIGIT PE/Fire 810, or anti‐HLA‐DR Spark Blue 574.

Tandem breakdown can produce variability in flow cytometry assays, which can be particularly problematic in high parameter panels. In Figure 6 we attempt to illustrate ways in which tandem breakdown can be dealt with in this context. The PE/Fire 810 tandem is relatively new, with a large Stoke's shift and high brightness that allows molecules stained with this fluorophore to be detected with relative ease. Not surprisingly, it is included in both the original 40‐color OMIP‐69 and the 50‐color OMIP‐102 panels. When dyes are first released, however, the variety of conjugates is generally quite limited, so there are few choices for panel design. In both panels, HLA‐DR was stained with PE/Fire 810. Note that we do not consider this to have been a poor choice and made the same choice ourselves for our work based on what was available at the time. This conjugate, however, is prone to differential breakdown, manifesting as higher breakdown (and more PE signal) on the monocytes as opposed to lymphocyte populations (Fig. 6). It can be difficult to achieve comparable levels of breakdown on the single‐stained controls as in the fully stained sample, resulting in unmixing error bias as well as spread.

We suggest that there are three main ways in which very labile tandems can be managed:

(1) Use the tandem stabilizer. Using the tandem stabilizer in this case reduced the percentage of cells appearing as PE^+^ monocytes (in the absence of any PE staining) by nearly half.
(2) Alter the panel design. Use labile tandems on lymphocytes, if possible. Switching the PE/Fire 810 to a target expressed on lymphocytes (TIGIT) at a lower level nearly ablates the appearance of false PE^+^ cells. This is because the tandem breakdown is catalyzed by the monocytes, likely through ROS production, and because the PE signal will be proportional to the PE/Fire 810 signal, so the lower expression of TIGIT compared to HLA‐DR sets an upper bound on how much false PE signal can appear. HLA‐DR can then be stained with a different dye that is not susceptible to tandem breakdown.
(3) Fix first. The tandem breakdown on monocytes occurs due to cellular activity that is quenched upon fixation. Staining post‐fix can largely abolish this unwanted breakdown, presumably through disruption of enzymatic activity.

Panel design is a rapidly shifting landscape due to rapid innovation in the field in both dye and cytometer technology. We in no way mean to disparage the accomplishments of the authors of OMIP‐69 or OMIP‐102 (Konecny et al., 2024; Park et al., 2020, 2024). OMIP‐69 has recently been updated to a second version (Park et al., 2024), which substitutes the anti‐HLA‐DR PE/Fire 810 to cFluor BYG750 due to concerns regarding stability.

**Figure 7:**
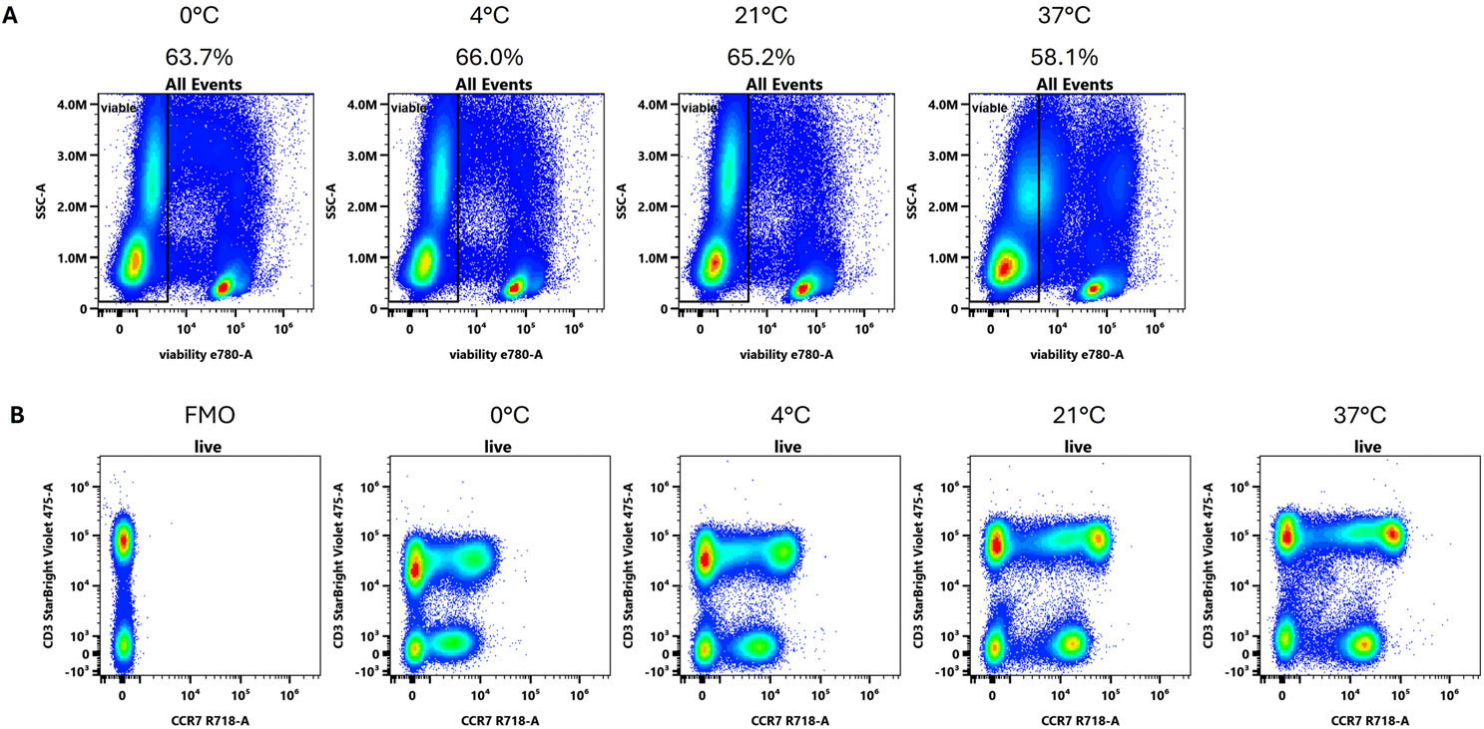
Impact of incubation temperature on sample viability and CCR7 detection. (**A**) Cryopreserved human PBMCs were stained for 1 hr at the indicated temperatures, and viability was assessed using the fixable viability dye eFluor 780. (**B**) Staining for CCR7 and CD3 on human PBMCs when the samples were incubated at the indicated temperatures. Staining was performed in FACS buffer directly after thawing. Example plots have been gated on single viable lymphocytes. The FMO shown was stained at 21°C.

Finally, while we have largely focused on reducing non‐specific binding, modulation of specific binding to increase signal can also be achieved by altering staining time or temperature (van Wolfswinkel et al., 2023; Whyte et al., 2022). Detection of chemokine receptors or antigen‐specific TCRs (via tetramer staining) can work better at higher temperatures, which can be due to recycling of molecules to the cell surface, increased fluidity of membrane‐proximal receptors allowing antibody access or simply the temperature‐dependence binding reaction as per the Gibbs free energy equation (Dolton et al., 2015; Johnstone et al., 1990; Schraml & von Proff, 2012; Voets, 2017). Higher temperatures can also invite staining artefacts via antibody capping, loss of viability or increases in non‐specific binding. In Figure 7, we illustrate the trade‐off in viability and staining intensity for cryopreserved human PBMCs, where increasing temperature during the incubation leads to enhanced CCR7 detection but also loss of cell viability, particularly among monocytes. In our high parameter panels, we have elected to use room temperature (i.e., 21°C) as the incubation temperature, which provides staining that is nearly as good as at 37°C while minimizing cell death. We note OMIP‐69 and OMIP‐102 also use room temperature staining. For samples derived from tissue digestion or other fragile sources, lower temperatures may be required.

### Troubleshooting

In Table 6, we aim to provide a quick guide for the user to start on the path to identifying and reducing non‐specific interactions in their flow cytometry.

**Table 6:** Troubleshooting Guide for Unwanted Signals

Problem	Possible cause	Solution(s)
Correlation between PE or APC and tandems thereof	Tandem breakdown	Always minimize light exposure
		Use tandem stabilizer
		Stain tandem after fixation
		Move tandem to a non‐myeloid cell type
		Move tandem to a lower expression marker
Correlation between SIRIGEN polymer dyes	Brilliant/Super Bright dye interaction	Use Brilliant Stain Buffer variants
		Minimize pre‐mixed time for Brilliant/Super Bright dyes
		Move Brilliant/Super Bright dyes to lower expression markers
		Swap to a non‐Brilliant/Super Bright dye
High background on myeloid cells	Inadequate blocking	Increase blocking time or concentration
		Pre‐block
		Test additional blocking reagents such as Monocyte Block
All cells are positive	Inadequate titration; non‐specific antibody	Re‐titrate
		Test alternate clone, especially with a different host species
		Try Fc‐mutated antibody
Poor separation	Can be caused by all antibody binding non‐specifically off‐target; usually caused by other factors, predominantly insufficient staining time, low temperature, or poor fluorophore pairing	Modify staining time
		Modify staining temperature
		Use a brighter fluorophore
		Investigate alternative clones

### Time Considerations

The addition of blocking steps adds a small amount of time to the staining workflows. In many cases, users performing surface staining only may observe comparable results by simply adding the blocking mix at the same time as the staining mix. For intracellular staining, we observe superior results with a separate pre‐blocking step.

The surface staining workflow is expected to take ∼2 hr, including the 1 hr staining incubation.

The overnight staining protocols require 2.5 to 3 hr time the first day, and 10 to 20 min the second day.

### Author Contributions

**Oliver Burton**: Conceptualization; formal analysis; investigation; methodology; validation; visualization; writing—original draft. **James Dooley**: Conceptualization; project administration; writing—review and editing. **Adrian Liston**: Conceptualization; funding acquisition; project administration; resources; supervision; writing—review and editing.

### Conflict of Interest

Oliver Burton provides flow cytometry consulting services and has consulted for Bio‐Rad, makers of StarBright dyes. The other authors declare no conflict of interest.

## Data Availability

Data including FCS files are available from the authors upon request.
